# The Palliative Outcome Scale (POS) applied to clinical practice and research: an integrative review

**DOI:** 10.1590/1518-8345.0993.2764

**Published:** 2016-08-15

**Authors:** Fernanda Capella Rugno, Marysia Mara Rodrigues do Prado De Carlo

**Affiliations:** 1 Doctoral Student, Escola de Enfermagem de Ribeirão Preto, Universidade de São Paulo, PAHO/WHO Collaborating Centre for Nursing Research Development, Ribeirão Preto, SP, Brazil.; 2 Professor, Faculdade de Medicina de Ribeirão Preto, Universidade de São Paulo, Ribeirão Preto, SP, Brazil.

**Keywords:** Palliative Care, Palliative Outcome Scale, Quality of life, Integrative Review

## Abstract

**Objective:**

to identify and evaluate the evidence found in the international scientific literature on the application of the Palliative Outcome Scale (POS) in clinical practice and research in Palliative Care (PC).

**Method:**

integrative literature review, through the search of publications in journals indexed in PubMed / MEDLINE, LILACS, SciELO and CINAHL databases, between the years 1999 and 2014.

**Results:**

the final sample consisted of 11 articles. In the data analysis, the articles were classified into 2 units of analysis (studies using the POS as a resource in research and studies using the POS in clinical practice), in which the information was presented in the form of sub-themes related to publications of the selected studies, highlighting the synthesis of the results.

**Conclusion:**

POS emerged as an important tool for measuring outcomes to assess the quality of life of patients and families, of the quality of care provided and the PC service organization. The international scientific literature on the application of POS proved to be relevant to the advancement and consolidation of the field of knowledge related to PC.

## Introduction

Among the clinical outcomes studied in oncology, evaluations of the survival curves and quality of life (QoL) are needed to direct the actions of health professionals^1^. However, to be reliable from a quantitative point of view, these assessments should be made using instruments to measure constructs that are valid, reliable and culturally adapted^2^ ^-^ ^3^.

Originally developed by a group of researchers from King's College London^4^, the Palliative Outcome Scale (POS) is a multi-dimensional assessment scale of QoL widely used, both in teaching and research as well as in clinical practice, applied in people suffering life-threatening chronic diseases in Palliative Care (PC)^1^. These are essential for the humane treatment to people with life-threatening clinical conditions whose treatment is not longer a modifier of the disease^5^.

The POS has two versions: the *self*, which is intended for patients with advanced disease, and the *proxy* for the health professional. Besides the fact of being directed to different subjects, the *proxy* version differs from the *self* because it has an additional item on the patient's clinical performance status (ECOG performance status).

In its two versions, the POS is a short scale consists of 11 items, easily applied, incorporating aspects of the physical and psychological symptoms, spiritual considerations, practical and psychosocial concerns. The answers are given in a Likert scale of 5 points, with the exception of item 9, which has 3 points, and one open question regarding the main problems experienced by the patient. The scores of POS range from zero to 40 points, being 0 a better QoL and 40, the worse QoL^6^ ^-^ ^8^.

The process of cultural adaptation and validation of POS has been completed in different countries and cultures in the following languages: Portuguese (of Portugal), Italian, Spanish (Spain and Argentina), German, French, Mandarin, Punjabi and Urdu. It is currently developing the validation of POS *self* version for the Brazilian Portuguese (POS-Br), which will enable the availability of the scale to be used as a data collection tool in scientific research and as a resource for clinical practice in the country^9^.

PC must be seen as one of the mainstays of comprehensive care treatment for people with advanced (and life-threatening) disease. However, in Brazilian culture, there is a shortage of specific assessment tools that can measure the importance of early referral to a PC service and its impact on QoL. In addition, the POS is an important tool for measuring outcomes that can foster the advancement of knowledge in PC, promote and optimize care in PC services and its results can help to minimize the suffering of patients with advanced disease.

This study is shaped as an integrative review, aiming to identify and evaluate the evidence found in international scientific literature, concerning the application of POS scale in clinical practice and research in PC. The following guiding question was the cornerstone of the integrative review: What are the available evidences in the literature regarding the impact of the use of POS in research and as a resource in clinical practice with patients in PC?

The evidence found in this study will enable researchers and health professionals to understand and acknowledge the importance of the use of POS in the treatment of patients with life-threatening diseases.

## Methodological Pathway

Through an integrative review, this study examined the scientific literature on the use of POS in the context of PC. This review followed the steps as suggested in the literature^10^ ^-^ ^13^: selection of the guiding question, definition of the eligibility criteria (inclusion and exclusion), defining the relevant information from the studies, evaluation of findings, interpretation and synthesis of the information found.

The literature survey of articles published in indexed journals was carried out in electronic databases: LILACS, SciELO, CINAHL and PubMed / MEDLINE. The criteria for inclusion of articles previously as defined for this review were: articles published in Portuguese (from Portugal), English and Spanish, between the years 1999 and 2014, with abstracts and available online full text in the selected databases (LILACS, SciELO, CINAHL and PubMed / MEDLINE). Articles of literature review were excluded (secondary data source) and those who had in their series population under 18 (since the POS was developed for use in adult patients) ^(^ ^4^.

 The descriptors "palliative care" (descriptor that encompasses the terms "hospice care" and "terminal care"), "Palliative Outcome Scale", "outcome assessment health care" and "quality of life" were combined via the Boolean connectors "AND" and "OR" in Portuguese and Spanish. It is worth mentioning that during the initial search, two records of integrative review were found, one of which addressed the POS validation studies^14^ and the other, the impact of APCA POS as a tool to improve patient care quality and their families^15^ (though, been secondary sources of data, these two studies were not eligible). 

**Figure 1:**
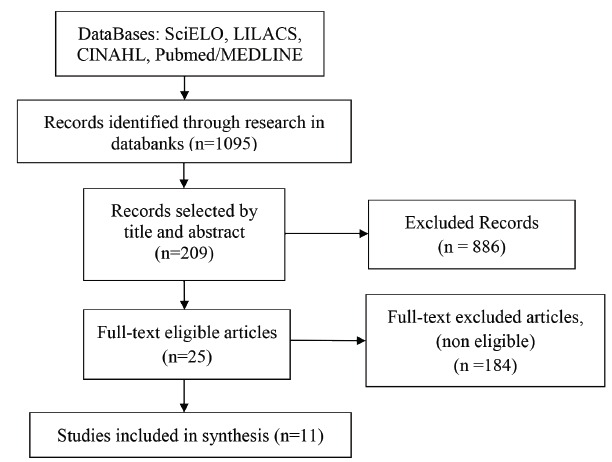
Search flow of the integrative review. Ribeirão Preto, SP, Brazil, 2015

The search identified 25 articles, of which 14 were excluded: 7 articles included translation, cultural adaptation and validation studies of the POS to other cultures, including the process of development and validation of the African Palliative Outcome Scale (APCA POS)^16^; 7 studies related to the use of APCA POS in institutions and research centers. At the end of the process, 11 studies on the use of POS in scientific research and clinical practice of PC teams were selected for the final sample of this integrative review. The process of search and selection of the material can be seen in Figure 1:

Using as a starting point the adaptation of an assessment instrument^12^, it was performed the synthesis of the included articles. Data collection captured the following information: year of publication; study title; name of the authors; journal of publication; instruments used and results found.

## Results

The consultation in 4 multidisciplinary databases and the findings ensured scientific and methodological rigor of the search (being itself representative of the international production). Among the articles included in the integrative review, 6 were published in magazines of the thematic area in PC, 3 in the thematic area of pain and other symptoms and 2 in the thematic area of QoL. The average impact factor of the journals is 2.402 (1.347-2.84).

Regarding the type of study design, the selected papers were: 5 methodological design studies ^17^ ^-^ ^21^ (3 studies tested the psychometric properties - "secondary analysis" and 2 development and validation studies of scale), 5 observational studies ^22^ ^-^ ^26^ (3 cross-sectional studies and 2 longitudinal studies) and one intervention study ^27^ (randomized controlled trial).

**Figure 2:**
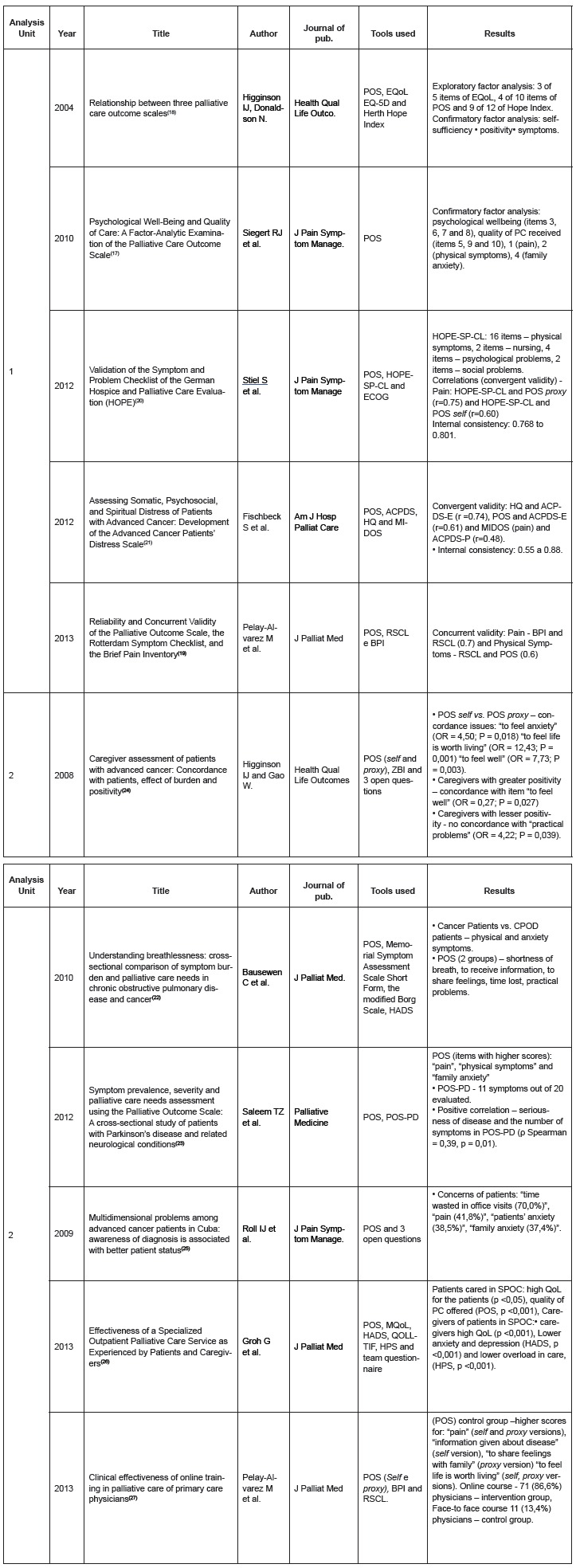
Synthesis of articles included in the integrative review - analysis units 1 and 2. Ribeirão Preto, SP, Brazil, 2015.

Figure 2 shows the information of the 11 papers.

The selected studies were classified into 2 units/categories of analysis: 1) Studies using the POS as a resource in research and 2) studies using the POS as a resource in clinical practice.

### Studies using the POS as a resource in research (analysis unit 1)

In this analysis unit, 5 studies^17^ ^-^ ^21^ with three subtopics were included: dimensions of POS (subtopic 1), comparison of the POS with other assessment tools (subtopic 2) and the use of POS for the development and validation new instruments (subtopic 3).

POS dimensions (subtopic 1): the research identified 2 main factors in POS^17^: one reflecting the extent of psychological well-being (items 3, 6, 7 and 8) and the other representing the quality of the PC received (items 5, 9 and 10). Confirmatory factor analysis proved that the POS is a multidimensional scale; according to the authors, patients in PC should be evaluated in a comprehensive manner (considering all aspects involving the welfare of the patient), and the ideal tools for this evaluation are multidimensional tools (e.g. POS).

POS comparison with other assessment tools (subtopic 2): it was verified the relationship of POS with the factor structure of the scales EuroQoL (EQ-5D) and the Herth Hope Index^18^. After correlation of the 3 scales and an exploratory factor analysis (EFA), the resulting selection is presented: 4 of the 10 items of POS ("pain", "sharing feelings", "to feel that life is worth living" and "to feel good"), 3 of 5 EQoL factors, and 9 of the 12 items of Hope Index; when it was made the EFA of the 3 combined scales, five factors / dimensions stood out. However, in the confirmatory factor analysis, 3 factors / dimensions appeared as relevant to the assessment for clinical practice: self-sufficiency (self-care, mobility, activities of daily living - ADL), positivity (sharing feelings and concerns, feeling good and valued) and physical symptoms.

The investigation of the concurrent validity and reliability of the POS, of the Rotterdam Symptom Checklist (RSCL) and the Brief Pain Inventory (BPI)^19^ revealed correlation between the intensity of the pain in the BPI and the pain in the physical scales of the RSCL, and between the physical symptoms of RSCL and those of POS; therefore "the physical scale of RSCL could be interchangeable for the symptoms of POS" and "pain intensity in BPI could be interchangeable for physical pain in the RSCL".

The use of POS for the development and validation of new instruments (subtopic 3): To validate the German Hospice and Palliative Care Evaluation (HOPE) Symptom and Problem Checklist (HOPE-SP-CL) ^20^, the domains of HOPE- SP-CL were correlated with the POS scores using the Spearman correlation coefficient (convergent validity). The results showed a strong correlation with the items related to symptoms in POS *proxy* and HOPE-SP-CL (r=0,75), as well as in POS *self* and HOPE-SP-CL (r=0,6). Psychometric properties showed that, as the POS, the HOPE-SP-CL is also a reliable and valid instrument.

The Advanced Cancer Patients' Distress Scale (ACPDS) ^(^ ^21^ is a screening tool of suffering / psychological distress for cancer patients in PC who are in the final stage of life. The ACPDS has 5 subscales: (1) "physical and emotional restrictions (ACPDS-E)" (2) "deficits in communication and information transfer (ACPDS-I)" (3) "the negative social reactions (ACPDS-N)" (4) "(fear of feeling) pain (ACPDS-P)", and (5)" gastrointestinal symptoms (ACPDS-G)". The test of the psychometric properties (convergent validity) of ACPDS was done with instruments Hornheide Questionnaire (HQ), Minimal Documentation System (MIDOS) and POS; significant correlations with the total score of POS were found (greater correlation between POS and subscale ACPDS-E; r = 0,61).

### Studies using the POS as a resource in clinical practice (analysis unit 2)

In this analysis unit, 6 studies ^22^ ^-^ ^27^ with 4 sub-themes were included: reviews of cancer and non-cancer patients (subtopic 1), *self* assessment ("patient-reported outcomes") versus *proxy* evaluation (perception of the caregiver) (subtopic 2), communication and improvement of PC team (subtopic 3) and *self* evaluation ("patient-reported outcomes") versus *proxy* evaluation (physician perception) (subtopic 4).

Reviews of cancer and non-cancer patients (subtopic 1): To compare the impact of the intensity of symptoms and patient's needs in PC in their overall survival, patients with metastatic cancer and patients with chronic obstructive pulmonary disease (COPD) were evaluated ^(^ ^22^. In addition to POS, it was applied the Memorial Symptom Assessment Scale short form (MSAS-SF), the modified Borg Scale and the Hospital Anxiety and Depression Scale (HADS). In general, the sum of the scores of POS was higher for patients with metastatic cancer than for patients with COPD. The average survival was 107 days for patients with metastatic cancer and 589 days for patients with COPD.

PC should also be offered in the early stages to patients with Parkinson's disease, multiple system atrophy or progressive supranuclear palsy^23^. Using the POS and Palliative Outcome Scale-Parkinson Disease (POS-PD) (a version adapted from the Palliative Outcome Scale-Symptom, POS-S), it is possible to check for various physical symptoms in this population: mobility problems in the lower limbs (51%), pain (39%), motion problems in upper limbs (28%), communication difficulties (28%), fatigue (24%), among others (for a total of 11 physical symptoms reported). 

Self assessment ("patient-reported outcomes") versus *proxy* evaluation (perception of the caregiver) (subtopic 2): To verify the correlation between the perception of the patient and primary caregiver in relation to QoL it was performed the assessment of 64 patients and 64 caregivers^24^ ; both groups completed the POS. Caregivers responded also to a burden overload scale, the Zarit Burden Interview (ZBI) and three open questions about the positive aspects of care. The results of this study showed greater concordance for the physical symptoms; the agreement was lower in relation to psychological symptoms and for the item "to feel that life is worth living" - and it was intensified when caregivers have high physical burden and little positivity about the act of caring.

Communication and improvement of the PC team (subtopic 3): One of the biggest problems for the PC teams in Latin America is the lack of communication (information failure) about the diagnosis and prognosis of patients with advanced cancer ^25^. The data analysis of 91 patients with advanced cancer in Cuba (data collection done through the POS plus 3 open questions - regarding the diagnosis and understanding of disease progression), pointed out a discrepancy between the information provided by the professionals and the information that patients wished for: only 41% were aware of the diagnosis; 59% would like to know about disease progression and clinical changes. In addition, POS showed that the aspects that bothered the patients more were "wasted time in office visits (70.0%)," "pain (41.8%)", the symptoms of "anxiety" (38.5%) and "family anxiety" (37.4%). 

To test the effectiveness and acceptance of specialized clinics in PC (SOPC - Specialized Outpatient Palliative Care)^26^, oncology patients answered questionnaires developed by the team (on physical symptoms), in addition to the standardized instruments POS and McGill Quality of Life Questionnaire (MQoL ). Caregivers also responded to the questionnaires developed by the team, along with the Hospital Anxiety and Depression Scale (HADS), the Quality of Life in Life-threatening Disease - Family Care Version (QOLLTI-F) and a short version of Häusliche Pflegeskala (HPS) (homecare scale). The results showed that, with the involvement of SOPC team, there was a significant improvement in quality of care and satisfaction with the care provided; the caregiver's burden was decreased and there was an increase in psychological support, as well as the performance in activities of daily living (ADLs). 

The *self* assessment ("patient-reported outcomes") versus *proxy* evaluation (physician perception) (subtopic 4): To measure the impact of online education in PC (Online palliative care education)^27^, general practitioners (primary care physician) were divided into 2 groups (66 in the intervention group and 58 in the control group). The intervention group had access to a program for online training (duration: 96 hours); and the control group had the option to voluntarily participate in a traditional training course in PC (classroom course of 20 hours). The intervention group had the participation of 63 patients and the control group of 54 patients. Physicians answered the POS *proxy* and the patients responded to the POS *self*, BPI and RSCL (in two different moments of the research). The results of comparing POS *proxy* with POS *self* showed "overestimation of psychological symptoms and information provided" and "underestimation of physical symptoms."

## Discussion 

This integrative review had as its main theme the implementation of POS in clinical practice and research in PC. It was carried out a careful evaluation of articles, focusing on the methodological procedure (instruments, data collection, analysis used), the main outcomes and limitations.

The analysis of the selected scientific literature has shown that the POS is a powerful QoL assessment tool for PC. Its application in clinical practice in PC can impact positively on improving the QoL of patients and families, improving quality of care, in the development and validation of other reliable instruments, in the organization of PC services and in the training of health professionals involved, in the early referral to PC (of both cancer and non-cancer patients) and in the improvement of the communication / integration of the patient-family/caregiver-professional triad. 

As shown in the analysis unit 1, as there are many scales currently under development, it is important to have a consensus on what scales should be used for each aspect/demand to be assessed. The choice of an instrument should take into consideration if it is valid and reliable ^(^ ^3^.

The maintenance / improvement of QoL is among the most expected outcomes in the PC services, and QoL assessments allow the appreciation of the information reported by the patients themselves ("patient-reported outcomes"). This perception of the patient is essential to guide the clinical practice of professionals ^(^ ^19^. Some domains of the questionnaires used to assess the symptoms and QoL in patients with advanced cancer can measure similar dimensions or constructs.

As there are few studies comparing the dimensions of instruments that have equivalent dimensions or constructs, an assessment of concurrent validity of these instruments may increase the knowledge of its psychometric properties and "interchangeability of equivalent areas"^17^ ^-^ ^19^. It is important to point out that this information was found in the subtopic 2 of unit 1. Nevertheless, the correlation between the areas of POS with EuroQoL scales (EQ-5D) and the Herth Hope Index^18^ may have been influenced by the previous knowledge that participants had in regard to their answers to the different scales (in addition, there was no change in the order of application of instruments). Regarding the POS, RSCL and BPI^19^, its limitations are related to the lack of results (statistically significant) to control for uncomfortable symptoms. Still, the outcomes of both studies are essential for the identification of the physical and psychosocial symptom of patients in PC.

The information on the dimensions of POS (subtopic 1) confirms it as the most assertive choice by teams of PC and research centers. Although the factor structure of POS has been verified by the exploratory and confirmatory factor analysis, the sample size was small; its factor structure also needs to be investigated with non-cancer populations.

The POS may be considered as the gold standard tool in the context of PC. Studies of unit 1 (subtopic 3) ^20^ ^-^ ^21^ reinforce its importance for the development and validation of new tools. Although the results found in the subtopic 3 were consistent (HOPE-SP-CL scale proved to be a reliable instrument - the Cronbach's alpha ranged from 0.768-0.801) ^(^ ^20^, this validation study was based on the analysis of secondary sources and its sample was restricted (made up of hospitalized cancer patients). Regarding the study of the development of ACPDS scale ^(^ ^21^, its study population should have been larger (the number of subjects is only three times larger than the number of items of the scale); the internal consistency of the scale ranged from 0.55 (less than the minimum acceptable) to 0.88 (a proper value). Despite the limitations already described, there was a high correlation between the POS and the HOPE-SP-CL, and also between POS and ACPDS (the correlation coefficients were higher than 0.6).

The use of POS can also contribute to the increase of the eligible patients to PC, whose target audience is still the cancer patients. The results found in Unit 2 (subtopic 1) proved the efficiency of this instrument for assessing QoL in non-oncologic patients and showed the need for QoL assessments - survival and multi-functional evaluations for patients with progressive neurological diseases ^23^ and COPD ^( 22)^. For patients with COPD, the predictors of survival could not be identified (the sample was small). For patients with progressive neurological diseases, the sample sub-represented those without autonomy and independence to participate in outpatient clinics, as well as those who had cognitive impairment (increased by dementia). When the *proxy* assessments are incorporated in addition to the *self* assessment, a greater number of patients with advanced stage disease may be included in research.

If the multi-functional assessments, QoL and overall survival (using validated instruments, in *self* and *proxy* versions) were part of routine care, they would promote the early referral of non-oncological populations to PC services ^(^ ^22^ ^-^ ^23^ as well as the issuing of specific guidelines.

For cancer patients, there are guidelines of the National Comprehensive Cancer Network (NCCN) ^(^ ^28^, suggesting as eligible for PC, patients with symptoms not under control or physical comorbidity and psychosocial relevant conditions (e.g, functionality according to the Karnofsky Performance Scale equal to or less than 50%, superior vena cava syndrome, spinal cord compression, cachexia, hypercalcemia, delirium, among others), as well as an estimated life expectancy of less than 12 months.

However, the PC continues to be underutilized even with cancer patients. Early referral to a PC service enables the evaluation, management and adequate relief of physical symptoms and psychological distress; moreover, it contributes to the discussions and planning of the end of life ^(^ ^29^.

Often these discussions and QoL assessments take into account only the perception of the caregiver (such as the progression of disease - not enabling the self-assessment of the patient). As it was presented in unit 2 (subtopic 2), the caregiver assessments were considered valid and reliable compared to patient assessments, especially when using the POS scale (scale considered easy to understand for both)^24^. However, the caregivers' burden and how they consider the care, can affect their understanding of the patient's QoL. It is also noteworthy that the analysis of the results did not allow identifying the real impact of the burden on the QoL of caregivers (sample size and number of variables not analyzed). It is necessary for clinicians to measure the caregivers' burden to verify and better interpret the information provided. The QoL of the caregivers, their support network and monitoring of the follow-up in the mourning period should also be addressed by the team^30^. 

Unfortunately, the number of PC services is still small compared to the incidence and prevalence of potentially fatal chronic degenerative diseases. All this is in favor of the idea of the implementation of SPOC around the world, as was pointed out by subtopic 3 of Unit 2; data collection (with application of the instruments in 2 different moments) for the study was performed only by a researcher, not a member of the SPOC team, and she was not blind to the answers of the evaluations. Nevertheless, the POS helped to measure how a specialized team can make a difference in the quality of life and ADL of patients and caregivers. After applying the POS, it could be seen an increase in the patient's QoL and relief of symptoms (especially pain - which is justified by the increased use of strong opioids)^26^. 

The use of POS can also identify gaps in the training of the team that staffs the services. The impact of the effectiveness of continuing medical education, in an online form on the clinical practice, has been scarcely studied (subtopic 4). Although online education has produced significant differences between groups regarding the knowledge of physicians in managing symptoms and improving communication, some confounding factors were diluted in the analysis (as the previous training received outside the study).

It is well known that education and development of capacities in PC (online or face-to-face) should be offered regularly to the medical and nonmedical staff. Through these activities, the team's attention would not be focused only on the patient's medical records, but also on his life story, desires and choices, resulting in greater agreement between "patient-reported outcomes" and *proxy* reviews ^(^ ^26^.

Communication skills should also be integrated and addressed in the courses. As evidenced in the subtopic 3, there is also an overestimation of the prognoses of patients in PC; this fact was even a limitation to the size of the sample (professionals had trouble in referring eligible patients for the study) ^(^ ^25^.

The results and main outcomes of this integrative review leave no doubt about the importance of POS and its impact on the holistic care of patients and families and the targeted and individualized clinical practice of the team. However, it should be noted that, precisely because it is a short scale, the results of POS should support further evaluation to investigate in greater depth each addressed aspect (physical and psychological symptoms, spiritual considerations, practical and psychosocial concerns).

Although POS contribute in the outcomes of the assessment of QoL, the scale should be used not only as an assessment tool, but as a clinical screening tool and as a means of transforming the current reality of the PC, substantiating real changes in the training of health professionals and assistance offered to patients and their families.

## Final considerations

Despite the growing knowledge in the field of PC, the research about its impact on QoL and related to comprehensive care for patients with life-threatening diseases, families and health teams, needs to consider concrete results based on clinical evidence. For these research results in PC to be significant and able to be incorporated into practical action, it is necessary to use tools or instruments that are reliable and valid assessment measures, such as POS. The POS is a scale of outcomes that incorporates in itself the multidimensionality of QoL in PC, and is characterized as a brief and simple tool for clinical application, feasible to be incorporated into the daily routine of professionals.

This integrative review helped to clarify the importance of POS, both for scientific research and for the optimization of clinical practice. The POS emerged as an important tool for the evaluation of the QoL of patients and families, the quality of care provided and the PC service organization. The international scientific literature on the use of POS proved relevant to the advancement and consolidation of knowledge in the PC field.

The evidence regarding the use of the POS as an evaluation tool, expands the understanding of health professionals and researchers about the importance of using outcome measures in evidence-based healthcare, promoting the early referral of patients with advanced disease to PC services, as well as enhances and strengthens the inclusion of the PC in public health policies in Brazil.

## References

[1] Wentlandt K, Krzyzanowska MK, Swami N, Rodin GM, Le LW, Zimmermann C (2012). Referral practices of oncologists to specialized palliative
care. J Clin Oncol.

[2] Luckett T, King MT, Butow PN, Oguchi M, Rankin N, Price MA (2011). Choosing between the EORTC QLQ-C30 and FACT-G for measuring
health-related quality of life in cancer clinical research issues, evidence and
recommendations. Ann Oncol.

[3] Pasquali L (2011). Psicometria: teoria dos testes na Psicologia e na Educação.

[4] Hearn J, Higginson IJ (1999). Development and validation of a core outcome measure for palliative
care the palliative care outcome scale. Palliative Care Core Audit Project
Advisory Group. Qual Health Care.

[5] Bausewein C, Fegg M, Radbruch L, Nauck F, Von-Mackensen S, Borasio GD (2005). Validation and Clinical Application of the German Version of the
Palliative Care Outcome Scale. J Pain Symptom Manage.

[6] Eisenchlas JH, Harding R, Daud ML, Pérez M, De Simone GG, Higginson IJ (2008). Use of the palliative outcome scale in Argentina a cross-cultural
adaptation and validation study. J Pain Symptom Manage.

[7] Serra-Prat M, Nabal M, Santacruz V, Picaza JM, Trelis J (2004). Validation of the Spanish version of the Palliative Care Outcome
Scale. Med Clin. (Barc).

[8] Aspinal F, Hughes R, Higginson IJ, Chidgey J, Drescher U, Thompson M. (2002). A user's guide to the Palliative care Outcome Scale..

[9] King's College London (2008). Questionnaires and Tools: Palliative Outcome Scale.

[10] Galvão CM, Sawada NO, Trevizan MA (2004). Systematic review a resource that allows for the incorporation of
evidence into nursing practice. Rev. Latino-Am. Enfermagem.

[11] Ursi ES, Galvão CM (2006). Perioperative prevention of skin injury an integrative literature
review. Rev. Latino-Am. Enfermagem.

[12] Mendes KDS, Silveira RCCP, Galvão CM (2008). Integrative literature review a research method to incorporate
evidence in health care and nursing. Texto Contexto Enferm.

[13] Santos CMC, Pimenta CAM, Nobre MRC (2007). The pico strategy for the research question construction and evidence
search Rev. Latino-Am. Enfermagem.

[14] Correia FR, De Carlo MMRP (2012). Evaluation of quality of life in a palliative care context an
integrative literature review. Rev. Latino-Am. Enfermagem.

[15] Dix O (2012). Impact of the APCA African Palliative Outcome Scale (POS) on care and
practice. Health Qual Life Outcomes.

[16] Harding R, Selman L, Agupio G, Dinat N, Downing J, Gwyther L (2010). Validation of a core outcome measure for palliative care in Africa the
African Palliative Outcome Scale. Health Qual Life Outcomes.

[17] Siegert RJ, Gao W, Walkey FH, Higginson IJ (2010). Psychological well-being and quality of care a factor-analytic
examination of the palliative care outcome scale. J Pain Symptom Manage.

[18] Higginson IJ, Donaldson N (2004). Relationship between three palliative care outcome
scales. Health Qual Life Outcomes.

[19] Pelayo-Alvarez M, Perez Hoyos S, Agra-Varela Y (2013). Reliability and current of the Palliative Outcome Scale, the Rotterdam
Sympt on Checklist and the Brief Pain Inventory. J Palliat Med.

[20] Stiel S, Pollok A, Elsner F, Lindena G, Ostgathe C, Nauck F (2012). Validation of the symptom and problem checklist of the German Hospice
and Palliative Care Evaluation (HOPE). J Pain Symptom Manage.

[21] Fischbeck S, Maier BO, Reinholz U, Nehring C, Schwab R, Beutel ME (2013). Assessing Somatic, Psychosocial, and Spiritual Distress of Patients
with Advanced Cancer Development of the Advanced Cancer Patients' Distress
Scale. Am J Hosp Palliat Care.

[22] Bausewein C, Booth S, Gysels M, Kuhnbach R, Haberland B, Higginson IJ (2010). Understanding breathlessness cross-sectional comparison of symptom
burden and palliative care needs in chronic obstructive pulmonary disease and
cancer. J Palliat Med.

[23] Saleem TZ, Higginson IJ, Chaudhuri KR, Martin A, Burman R, Leigh PN (2013). Symptom prevalence, severity and palliative care needs assessment
using the Palliative Outcome Scale a cross-sectional study of patients with
Parkinson's disease and related neurological conditions. Palliat Med.

[24] Higginson IJ, Gao W (2008). Caregiver assessment of patients with advanced cancer concordance with
patients, effect of burden and positivity. Health Qual Life Outcomes.

[25] Justo Roll I, Simms V, Harding R (2009). Multidimensional problems among advanced cancer patients in Cuba
awareness of diagnosis is associated with better patient status. J Pain Symptom Manage.

[26] Groh G, Vyhnalek B, Feddersen B, Fuhrer M, Borasio GD (2013). Effectiveness of a specialized outpatient palliative care service as
experienced by patients and caregivers. J Palliat Med.

[27] Pelayo-Alvarez M, Perez-Hoyos S, Agra-Varela Y (2013). Clinical effectiveness of online training in palliative care of
primary care physicians. J Palliat Med.

[28] National Comprehensive Cancer Network (2011). Clinical Practice Guidelines in Oncology. Palliative Care.

[29] Wentlandt K, Krzyzanowska MK, Swami N, Rodin GM, Le LW, Zimmermann C (2012). Referral practices of oncologists to specialized palliative
care. J Clin Oncol.

[30] Schofield HL, Murphy B, Herrman HE, Bloch S, Singh B (1997). Family caregiving measurement of emotional well-being and various
aspects of the caregiving role. Psychol Med.

